# Double congenital abnormalities of left anterior descending artery: a technical modification for closing wide neck of aneurysm and fistulae—a case report

**DOI:** 10.1093/ehjcr/ytaf263

**Published:** 2025-05-26

**Authors:** Rengin Çetin Güvenç, Abdullah Ayar Al Arfaj, Demet Doğan, Nihat Özer

**Affiliations:** Department of Internal Medical Sciences, Division of Cardiology, Istanbul Okan University School of Medicine, Tepeören Mahallesi Tuzla Kampüsü, Tuzla/Istanbul 34959, Turkey; Department of Internal Medical Sciences, Division of Cardiology, Istanbul Okan University School of Medicine, Tepeören Mahallesi Tuzla Kampüsü, Tuzla/Istanbul 34959, Turkey; Department of Internal Medical Sciences, Division of Radiology, Istanbul Okan University School of Medicine, Tepeören Mahallesi Tuzla Kampüsü, Tuzla/Istanbul 34959, Turkey; Department of Internal Medical Sciences, Division of Cardiology, Istanbul Okan University School of Medicine, Tepeören Mahallesi Tuzla Kampüsü, Tuzla/Istanbul 34959, Turkey

**Keywords:** Coronary fistulae, Coronary artery aneurysm, Coil embolization, Percutaneous intervention, Case report

## Abstract

**Background:**

The duplication of the left anterior descending coronary artery and coronary artery-to-pulmonary artery fistulae are infrequent congenital anomalies described in literature. These anomalies can lead to life-threatening conditions such as myocardial infarction, rupture, cardiac tamponade, and heart failure.

**Case summary:**

A 73-year-old male with chronic kidney failure was admitted for a preoperative cardiovascular assessment. Initially, he did not report any chest pain. However, while awaiting myocardial perfusion scintigraphy, he developed chest pain and coughing, leading him to present to the emergency department, where his troponin levels were found to be elevated. Coronary angiography and coronary computed tomography angiography showed a dual left coronary artery where one of the left anterior descending arteries completely transforms into a congenital aneurysm and fistula. In order to reshape the aneurysm neck and prevent the migration of coils into the left main coronary artery by creating a landing zone and to reduce the number of coils and the procedure time, two stents were first placed inside the aneurysm neck. Then, the fistula and aneurysm were successfully closed by coil implantation.

**Discussion:**

Some case studies and centre experiences recommend interventional closure using cover stents, vascular plugs, and coil embolization techniques for symptomatic fistulae and those resulting in complications. Despite these recommendations, determining the best treatment strategy remains challenging due to the lack of clear guidelines. The novel modified technic consisted of two nested stents and coil embolization to close the aneurysm and fistula and prevent secondary complications due to myocardial infarction.

**Learning points**

- Coronary artery fistulae can precipitate myocardial ischaemia as the flow is diverted from the main coronary branch. Up to 20% of all coronary fistulae are accompanied by an aneurysm on the fistula tract, with the additional hazard of aneurysm rupture.
- Implanting a covered stent across the ostium of the fistula is the conventional method to occlude a fistula percutaneously, but this approach risks leaving a stent in the main branch that can act as a focus for stent thrombosis or restenosis.
- We introduce a new modification of the stent-assisted coil embolization technique, where stents serve as a landing zone for the coils that are deployed to close the fistula tract and the neck of aneurysm simultaneously.

## Introduction

Congenital anomalies of the coronary arteries may involve differences in their number or location, as well as the presence of fistulae and aneurysms. These issues can lead to serious complications, including myocardial infarction, sudden death, and cardiac tamponade. In this case, a combination of these two conditions that resulted in a complex myocardial infarction was observed. We introduced a new method that was developed by modifying existing interventional techniques to effectively close the fistulae and aneurysms.

## Summary figure

**Figure:**
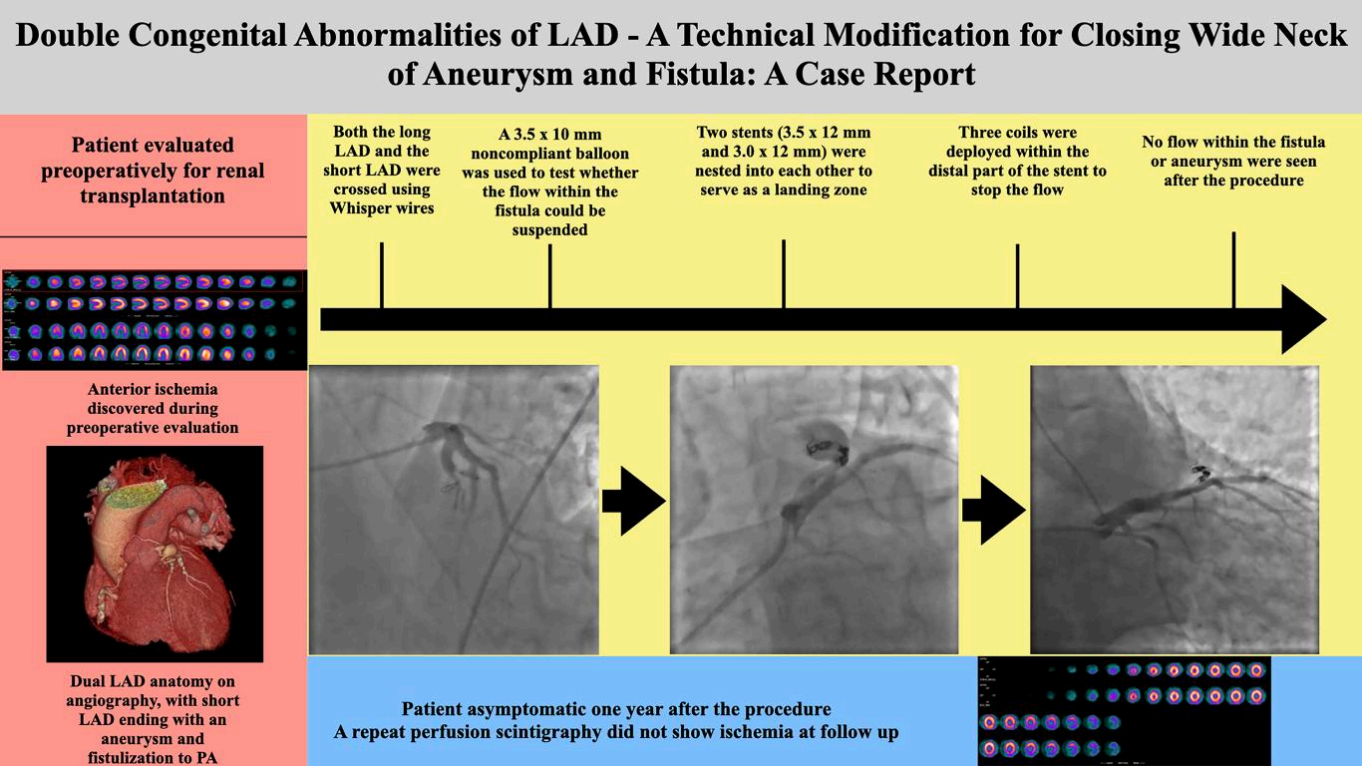


## Case presentation

**Figure 1:**
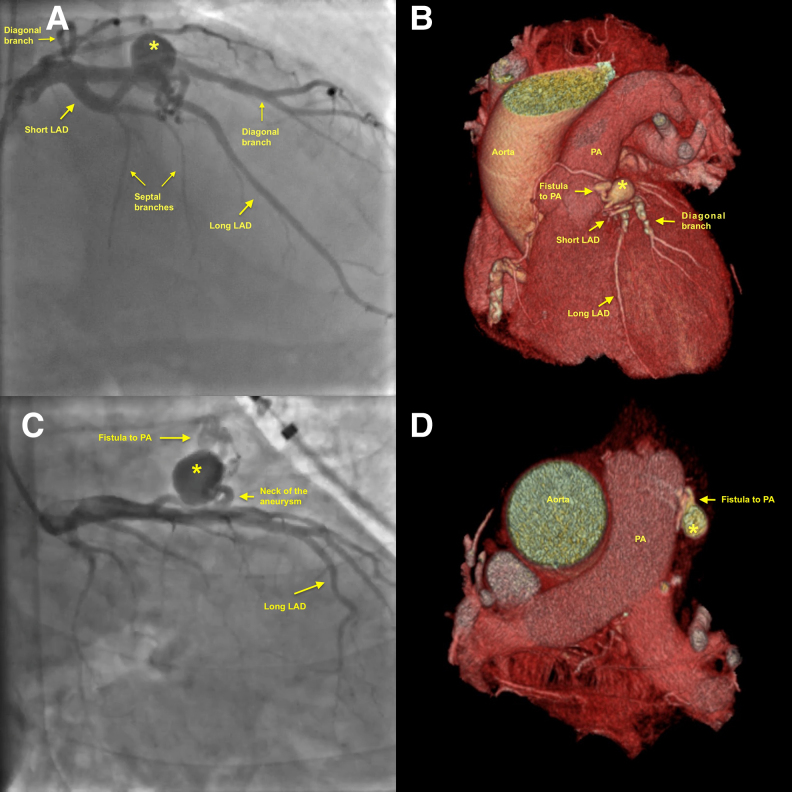
Angiographic images showing the short and long left anterior descending arteries and the aneurysm from the right anterior oblique cranial projection (*A*) with matching 3D computed tomography angiographic view showing the same structures (*B*). Right anterior oblique caudal projection allowed better visualization of the aneurysm neck, aneurysmal sac, and fistula to the pulmonary artery (*C*). Horizontal cross-sections at the level of pulmonary artery show matching images of fistula and aneurysm on T angiography (*D*). Asterisk shows the aneurysmal sac. LAD, left anterior descending artery; PA, pulmonary artery.

**Figure 2:**
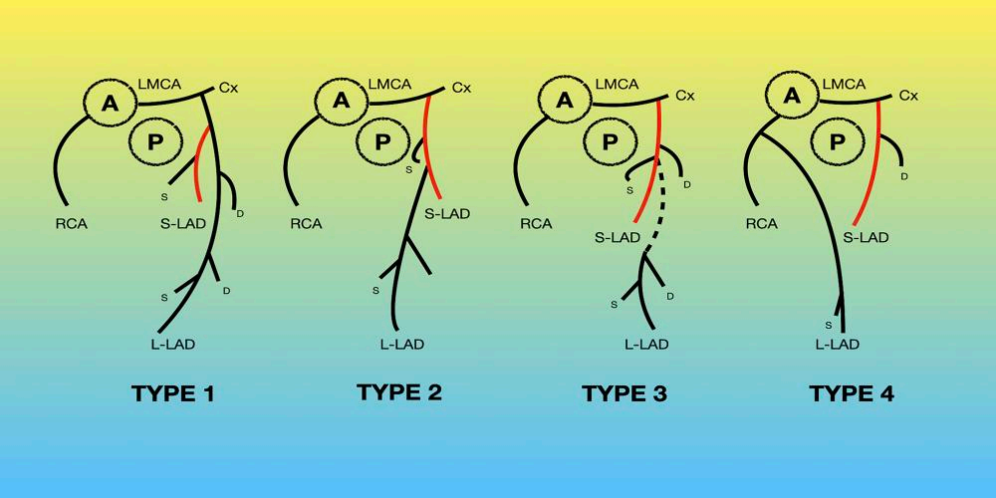
Spindola-Franco classification of the dual left anterior descending artery anatomy. The patient had a Type 1 dual left anterior descending artery anatomy per this classification. A, aorta; Cx, circumflex artery; D, diagonal artery; LMCA, left main coronary artery; L-LAD, long left anterior descending artery; P, pulmonary artery; S, septal arteries; S-LAD, short left anterior descending artery.

**Figure 3:**
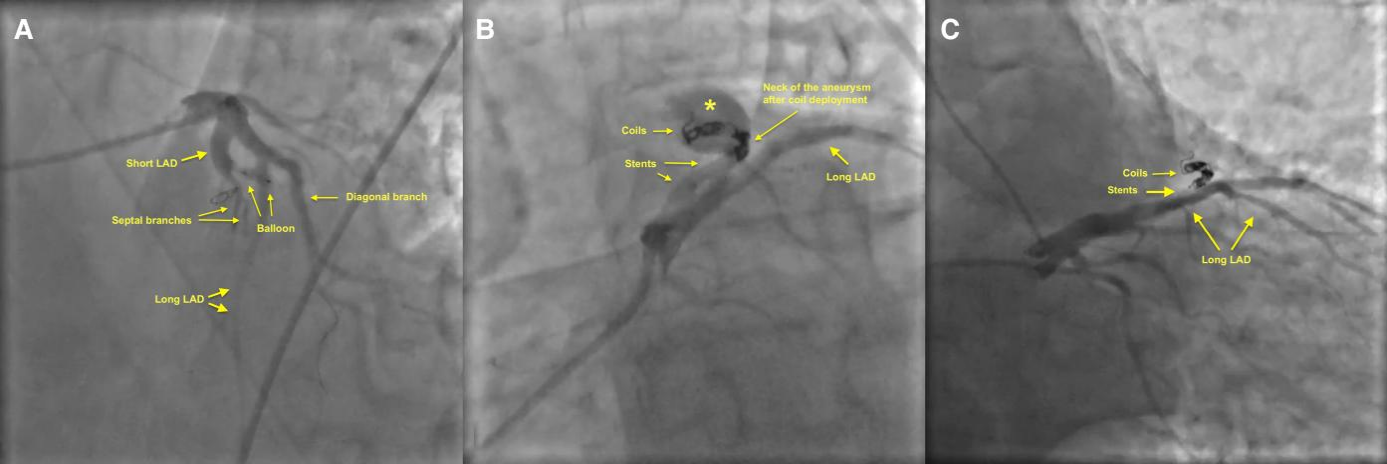
Angiographic images showing the intraprocedural steps for the closure of the aneurysm and the fistula. Initially, both the short and long left anterior descending arteries were wired using hydrophilic coated wires. A non-compliant balloon was then inflated at the neck of the aneurysm to understand whether the aneurysm and fistula could be closed via occluding this section and to determine the size needed for stents (*A*). A larger stent was then implanted to the proximal part of the neck, which is followed by a second smaller stent to narrow the neck. A microcatheter was then advanced to deploy the three coils to the distal part of the stent to occlude the neck of the aneurysm (*B*). Final angiographic image showed complete occlusion of the aneurysm and fistula, with coils seen at the neck of the aneurysm protruding into the aneurysmal sac (*C*). LAD, left anterior descending artery.

**Figure 4:**
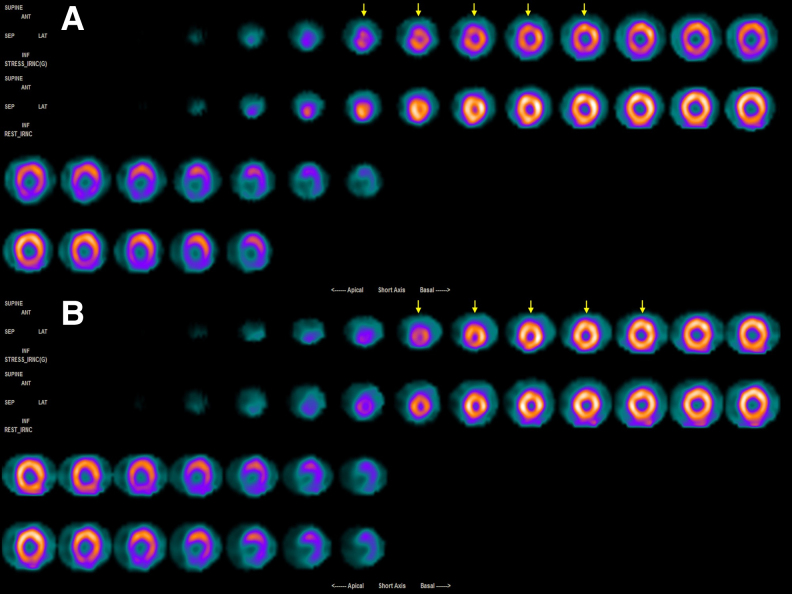
Myocardial perfusion scintigraphy images showing anterior ischaemia before the procedure (*A*) and normal perfusion following the procedure (*B*). Arrows on both panels point to the anterior left ventricular wall, where exercise-induced ischaemia can be seen on preoperative (*A*) but not on the postoperative (*B*) images.

A 73-year-old renal transplant candidate was admitted to our outpatient clinic for preoperative cardiovascular assessment. His medical history includes a kidney transplant that was performed 15 years ago. However, the kidney failed again 2 years ago, and he started undergoing haemodialysis three times a week. The patient did not have any cardiac complaints. Physical examination was unremarkable. Electrocardiography (ECG) showed pathologic Q wave and T wave inversion on leads V1 and V2. Echocardiogram revealed ascending aortic dilatation and mildly calcified aortic valve. The patient was referred to myocardial perfusion scintigraphy (MPS). Two days later, while waiting for the MPS result, the patient presented to the emergency department with retrosternal chest pain and coughing. There were no remarkable ECG changes at presentation, but troponin elevated progressively to a maximum of 3.61 ng/mL (upper limit of normal 0.1 ng/mL), and a thorax computed tomography (CT) showed bilateral consolidation of the lower lungs. He was diagnosed with Type 2 myocardial infarction secondary to pneumonia and subsequently hospitalized. As his scintigraphy results were compatible with mild-to-moderate ischaemia in the anterior wall, an early elective coronary angiography was planned following the resolution of pneumonia. The angiogram showed an ectatic right coronary artery, a thin circumflex left coronary artery, and a dual left anterior descending artery (LAD) morphology. Several small septal arteries emerged from the short LAD, and the remainder of the artery fistulized into the pulmonary artery (*Figure 1A–D*; *Video 1*). A crab-like saccular aneurysm was present on the fistula tract (*Figure 1A–D*; *Video 1*; Supplementary material online, *Video S1*). No significant lesions were observed on the long LAD that perfused the majority of the anterior wall. To delineate coronary anatomy and to plan the intervention, we decided to perform coronary CT angiography. The coronary CT angiography results confirmed that the patient had dual LAD (Type 1 per Spindola-Franco classification) (*Figure 2*),^1^ with the short LAD having a saccular aneurysmatic enlargement of 20 × 15 mm and a fistula that connected the aneurysm to the pulmonary artery (*Figure 1A–D*). The case was then reviewed in a heart team meeting, where we agreed to proceed with a percutaneous intervention due to elevated surgical risk. After wiring both LADs, we inflated a 3.5 × 10 mm non-compliant balloon at the aneurysm neck to assess whether closing the neck could stop the flow within the aneurysm (*Figure 3A*; Supplementary material online, *Video S2*). Subsequently, we have implanted a stent that matched the reference vessel diameter (3.5 × 12.0 mm Supraflex™) and a smaller stent within the first stent (3.0 × 12.0 mm Biotronik AG) to create a landing zone within the neck of the aneurysm that would narrow the neck and minimize the risk of coil migration (*Figure 3B*; Supplementary material online, *Videos S3* and *S4*). Following creation of this landing zone, three coils (2.0 × 4.0 mm, 3.0 × 12.0 mm, and 2.0 × 4.0 mm, Boston Scientific Interlock) were deployed via a microcatheter into the distal part of the stents (Rebar, Medtronic, USA) (*Figure 3B* and *C*; *Video 2*). Final contrast injections showed that the aneurysm, the small arteries it connects, and the fistula were completely closed (*Figure 3C*; *Video 3*). The patient was discharged 1 day after the procedure with 100 mg aspirin and 20 mg atorvastatin. During the 6-month, 1-year follow-ups, he reported no complaints. The exercise test showed good functional capacity. At 1-year follow-up, a repeat scintigraphy showed no sign of ischaemia (*Figure 4A* and *B*).

## Discussion

Dual LAD is an infrequent congenital coronary anomaly that is seen in up to 6% of coronary angiographies.^2^ To the best of our knowledge, this is the first case in the literature where the continuation of one of the dual LADs is transformed into an aneurysm and ultimately fistulizes into the pulmonary artery. Congenital coronary artery fistulae (CAFs) typically form between the coronary artery and either the right ventricle or the pulmonary artery.^3^ The incidence of coronary artery aneurysm (CAA) and fistula coexistence is estimated as 20%, but good quality evidence is still unavailable.^4^ In the present case, the fact that the aneurysmatic segment is separated by a wide root and that the entire short LAD has turned into an aneurysm ending a fistula and has no functional branches is compatible with a congenital aetiology. The myocardial injury that we have observed in our patient is probably related to the steal phenomenon triggered by the increased metabolic stress related to pneumonia and chronic kidney failure.

To treat complications of fistulae, such as myocardial ischaemia, infarction, and sudden death despite based on limited case series, two methods are suggested: percutaneous intervention or surgery.^3,5–9^ Percutaneous closure techniques involve using embolization coils, vascular occluders, and stent grafts.^10^ Coil embolization is preferred in small fistulae where tortuosity is severe.^4^ The use of stent grafts is limited to complex fistulae to preserve the patency of the parent vessel where the anatomy is suitable,^11,12^ but they are associated with low procedural success, a high risk of no reflow, and unacceptable rates of early and late recurrent myocardial infarction.^3,9,13–16^ A study showed that the risk of stent graft thrombosis and related myocardial infarction was 16.5% in a 1-year period.^13^ Stent-assisted coil embolization, where coils are placed on the exterior surface of a stent, can only be done when there is no associated fistulae and even a large number of coils are needed to close the aneurysm.^9,14^

In the present case, the primary challenge that we faced was the possibility of coil migration due to the wide neck of the aneurysm that precluded using a coil-only technique and the potential for late obstruction of the long LAD in case a stent graft is preferred. In contrast to established techniques, where a covered stent is used to isolate the aneurysm from the parent artery or coils are deployed to the exterior surface of an uncovered stent to fill the aneurysmal sac, we used two stents placed into one another to create a landing zone that constricted the aneurysm neck and deployed coils to the inner side of these stents to suspend blood flow through the neck of the aneurysm (*Figure 3A–C*). This approach effectively suspended blood flow to both the aneurysm and fistula using fewer coils, since coils served as an occluder anchored to a stent rather than being used as a means to fill the aneurysmal sac (*Figure 3C*).

Main alternatives for the management of the present case would be either conservative management, surgical correction of the fistula and aneurysm, or implanting a covered stent to the long LAD across the ostium of the short LAD. Conservative management would not ameliorate the risk for aneurysm rupture, and it is uncertain to what degree ischaemia could be reduced when the cause is not related to atherosclerotic disease. For the surgical approach, the patient was discussed in a heart team meeting and he was deemed to have a high risk for a cardiovascular surgical procedure given that he had end-stage renal failure. For the covered stent approach, a covered stent implanted on the long LAD across the ostium of the short LAD would occlude septals originating from the short LAD, in addition to occluding other major branches (including one diagonal artery) originating from that segment (*Figure 1A*). Also, a covered stent on the long LAD would serve as a potential nidus for thrombosis and restenosis, as aforementioned.

Some studies found that the long-term risk of recurrence varies between 9% and 19% for patients treated with transcatheter closure methods and up to 25% for those undergoing surgical closure.^17^ Although it is not possible to predict long-term recurrence risk associated with the presently described method, we consider that the presence of stents and coils within the neck of the aneurysm would promote development of neointimal proliferation and hasten complete closure of the aneurysm and the fistula. Indeed, we did not observe ischaemia on the repeat perfusion imaging 1 year after the original procedure, which indicates that there was no haemodynamically significant flow diversion within the LAD. However, long-term safety and reliability of this method should be tested in future studies.

## Conclusion

There is yet to be an optimal strategy for managing CAAs and fistulae, due to their rarity as well as lack of controlled studies and trials on this subject. In the absence of evidence, it is reasonable to decide on strategies on an individualized basis, depending on the morphology and location of the CAA and CAF. The present approach had a couple of advantages over previous methods described in the literature, namely reducing the number of needed coils while simultaneously not risking the patency of the parent vessel. This novel technique should be technically feasible for most patients, but further work is needed to establish the safety and reliability of this method.

## Supplementary Material

ytaf263_Supplementary_Data

## Data Availability

The data underlying this article will be shared on reasonable request to the corresponding author.

## Lead author biography

Rengin Çetin Güvenç is currently an associate professor at the Department of Cardiology of Okan University Hospital in Turkey. Her research and clinical area include adult congenital heart disease, heart failure, and multimodality cardiac imaging. She a member of adult congenital heart disease group of the Turkish Society of Cardiology and the European Society of Cardiology.
